# Genetic insights into MIS-C Post-COVID-19 in Kuwaiti children: investigating monogenic factors

**DOI:** 10.3389/fcimb.2024.1444216

**Published:** 2025-01-08

**Authors:** Mohammed Dashti, Hessa AlKandari, Md Zubbair Malik, Rasheeba Nizam, Sumi Elsa John, Sindhu Jacob, Arshad Channanath, Fouzeyah Othman, Safa Al-Sayed, Osama Al-Hindi, Mona Al-Mutari, Thangavel Alphonse Thanaraj, Fahd Al-Mulla

**Affiliations:** ^1^ Department of Genetics and Bioinformatics, Dasman Diabetes Institute, Kuwait City, Kuwait; ^2^ Department of Population Health, Dasman Diabetes Institute, Kuwait City, Kuwait; ^3^ Department of Pediatrics, Farwaniya Hospital, Ministry of Health, Farwaniya, Kuwait; ^4^ Department of Paediatrics, Sabah Hospital, Ministry of Health, Kuwait City, Kuwait; ^5^ Department of Paediatrics, Adan Hospital, Ministry of Health, Ahmadi, Kuwait

**Keywords:** multisystem inflammatory syndrome in children, coronavirus infection, MIS-C, exome sequencing, monogenic

## Abstract

**Background:**

Multisystem inflammatory syndrome in children (MIS-C) is a severe complication arising from SARS-CoV-2 infection, with indications that rare inborn errors of immunity may play a role in its pathogenesis. Recent studies suggest that genetic predispositions, particularly monogenic forms, could significantly influence the immune responses to SARS-CoV-2 in MIS-C.

**Methods:**

We analysed 24 children under 12 years old, all of whom met the criteria provided by the World Health Organization, 2020 for MIS-C diagnosis, from the Paediatric COVID-19 Registry in Kuwait (PCR-Q8). Demographic and clinical data were collected from medical records, and exome sequencing was performed on the children and their parents to identify rare exonic variants. These variants were prioritized using two approaches: a candidate genes approach employing trio segregation analysis, and a candidate variants approach using a gene panel informed by previous studies on MIS-C-related genetic variants and datasets of differentially expressed genes in MIS-C patients.

**Results:**

The candidate genes approach identified 53 unique genes in 20 of the 24 probands, including *DDX60* and *TMEM154*, which were also differentially expressed between MIS-C and control groups. The candidate variants approach identified 33 rare, predicted deleterious heterozygous variants across 19 unique genes in 19 of the 24 probands, including both previously described and novel candidate variants for MIS-C. Pathway analysis of the identified genes from both approaches revealed significant involvement in immune response, viral defence, and inflammatory pathways.

**Conclusion:**

This study underscores the monogenic susceptibility to MIS-C, enhancing the evidence base through comprehensive genetic analysis. The findings highlight the critical role of genetic predispositions in MIS-C and suggest that further functional genomics work is necessary to explore the mechanistic contributions of these genes, facilitating the development of targeted diagnostic strategies.

## Introduction

Multisystem inflammatory syndrome in children (MIS-C) is a serious complication associated with current or recent SARS-CoV-2 infection or COVID-19 exposure, typically occurring weeks after the initial infection. MIS-C was first identified in April 2020 in regions with high COVID-19 rates across Europe, North, and South America (Whittaker et al., 2020; Verdoni et al., 2020; Toubiana et al., 2020; Riphagen et al., 2020; Jones et al., 2020; de Farias et al., 2020). A recent study reported MIS-C cases in the Middle East, specifically in the United Arab Emirates and Jordan (Abuhammour et al., 2022).

Population-based incidence estimates for MIS-C indicate approximately 316 cases per million SARS-CoV-2 infections (Payne et al., 2021). Incidence rates vary by ethnicity, race, and pandemic phases. A U.S. study reported higher incidence among Black, Hispanic or Latino, and Asian or Pacific Islander populations compared to whites. In Israel, MIS-C incidence per 100,000 persons under 18 years was 54.5 during the Alpha, 49.2 during the Delta, and 3.8 during the Omicron waves (Levy et al., 2022). In Cape Town, South Africa, the incidence was 22 per 100,000 exposures, with black children over-represented in MIS-C cases. In the U.S., MIS-C incidence after vaccination was as low as 1.0 case per million individuals receiving one or more doses of any COVID-19 vaccine in the age group with a median age of 16 years (Yousaf et al., 2022).

Epidemiological data suggest that SARS-CoV-2 infection triggers MIS-C onset, typically about one month post-infection. MIS-C is a heterogeneous condition resembling Kawasaki disease (KD), a clinically diverse paediatric inflammatory disorder (Verdoni et al., 2020). It has been proposed that rare inborn errors of immunity (IEIs) can modify the immune response to SARS-CoV-2, contributing to MIS-C pathogenesis in some children (Sancho-Shimizu et al., 2021). Discovering monogenic IEIs underlying MIS-C could provide insights into its pathogenesis. Chou et al. (2021) found monogenic susceptibility to inflammation in their MIS-C patient cohort. Investigations into rare recessive genetic variants have linked severe SARS-CoV-2 outcomes to defects in genes involved in type I interferon response (Zhang et al., 2020). The Middle Eastern study (Abuhammour et al., 2022) identified rare, likely deleterious heterozygous variants in immune-related genes, including *TLR3, TLR6, IL22RA2, IFNB1*, and *IFNA6*, in 19 of 45 MIS-C patients, with 7 patients carrying multiple variants.

Given the rarity of inflammatory disorders and the genetic predisposition observed in MIS-C patients (Chou et al., 2021), our study aims to further elucidate monogenic susceptibility to MIS-C. We analysed a cohort of family trios of MIS-C patients from Kuwait, focusing on familial segregation of autosomal recessive, X-linked recessive, and *de novo* mutations. Using exome sequencing, we identified ultra-rare and potentially damaging variants, highlighting novel candidate genes for MIS-C potentially unique to the Middle Eastern population. We employed stringent criteria and methodologies used by other research groups to prioritize variants in known MIS-C associated genes (Abuhammour et al., 2022; Vagrecha et al., 2022; Reis et al., 2023; Lee et al., 2023). By integrating these approaches, we aim to deepen our understanding of the genetic basis of MIS-C and contribute to the development of targeted therapeutic strategies.

## Materials and methods

### Study cohort

Sixty-seven children aged ≤ 12 years who met the MIS-C diagnosis criteria provided by the World Health Organization, 2020 (World Health Organization, 2020) were identified, during April 2020 and October 2021, from the national Paediatric COVID-19 Registry in Kuwait (PCR-Q8) (Qabazard et al., 2022). The criteria for diagnosis of MIS-C included age ≤12 years, fever ≥38.0°C, positive SARS-CoV-2 RT-PCR or positive antibody or positive antigen test. Multisystem involvement was shown by two or more categories involving (i) Kawasaki disease (KD) or toxic shock syndrome, (ii) rash (bilateral non-purulent conjunctivitis, or mucocutaneous inflammation signs), (iii) gastrointestinal involvement (abdominal pain, vomiting, or diarrhoea) and (iv) cardiovascular involvement (elevated troponin or Left Ventricular Ejection Fraction (LVEF) <55% or Coronary Artery (CA) dilation, aneurysm, or ectasia on echocardiogram).

Detailed demographic and clinical phenotype data were obtained from medical charts. Out of the 67 children initially diagnosed with MIS-C and included in the study, exome sequencing was performed on 24 children for whom DNA samples from both parents were available. This formed 24 trios, which were analysed as part of this study (**Supplementary Table 1**).

The study protocol was approved by the Ethical Review Committee at Dasman Diabetes Institute and the Ethical review Committee at the Ministry of Health of Kuwait and was in accordance with guidelines of the Declaration of Helsinki, and of the United States Federal Policy for the Protection of Human Subjects. Prior to participating in the study, every adult participant signed the informed consent form. As regards paediatric subjects, informed consent was obtained from the parents/legal guardians and assent was obtained from children aged 7 years or more.

### DNA extraction

Blood samples were collected in ethylenediaminetetraacetic acid (EDTA)-treated tubes and genomic DNA was extracted using a QIAamp Blood DNA kit (Qiagen, Germany). A Qubit Fluorometer (Thermofisher, USA) was used to quantify spectrophotometry according to the manufacturer’s protocol. Absorbance values at 260–280 nm was checked for adherence to an optical density range of 1.8–2.1.

### Exome sequencing

Exome sequencing of 72 individuals (24 trio families) was performed on the Illumina NovaSeq 6000 platform using Illumina DNA Prep with Enrichment kit (Illumina, CA, USA). Burrows wheeler aligner tool (Li and Durbin, 2009) was used to map short paired-ends to human Genome Reference Consortium Human Build 37 (GRCh37). Sequence Alignment Map tool (Li et al., 2009) was used to convert mapped data to compressed binary version (BAM) and PCR duplicate reads were marked using Picard software (http://picard.sourceforge.net). Variant calling was performed using the Genome Analysis Toolkit (GATK) (McKenna et al., 2010) on multiple Genomic Variant Call Format (GVCF) files generated for all samples after performing local realignments of reads that overlapped with insertion or deletion of bases (INDEL) as well as recalibration of base quality. Subsequently, a single Variant Calling Format file (VCF) was generated using GATK, which was later subjected to hard filtering parameters that include (“QD < 2.0”, “FS > 60.0”, “MQ < 40.0”, “MQRankSum < -12.5”, and “ReadPosRankSum < -8.0”) for single nucleotide variant (SNV) and (“QD < 2.0”,”FS > 200.0”, and “ReadPosRankSum < -20.0”) for INDEL. Variants that pass quality filtering were annotated and functionally categorized using ANNOVAR tool (Wang et al., 2010) version de74a7d59955d769c6cbb92a0d64d12c90c8eede.

#### Approach 1: MIS-C monogenic candidate genes prioritization workflow

We analysed the entire exome data (~20,000 genes) of MIS-C-afflicted children to identify genetic variants for further prioritization. High-quality variants were annotated for functionality using ANNOVAR (Wang et al., 2010). Only functional types of variants, such as missense, loss-of-function, stop-gain/loss, and splice-site mutations, were considered. These variants were then examined for global frequencies reported in ExAC (version hg19_r0.2), 1000 Genomes Project (phase1 release v3), and gnomAD exome (version 4.0) databases, retaining only ultra-rare variants with a minor allele frequency (MAF) of ≤0.01%.

To evaluate pathogenicity, we used the Combined Annotation Dependent Depletion (CADD) score via the Ensembl Variant Effect Predictor (VEP: McLaren et al., 2016), applying a Phred-like score cut-off of 20 (top 1% of deleterious variants in the human genome). In the absence of controls from siblings or extended family members, candidate variants were analysed for segregation within the family based on specific modes of inheritance, including autosomal recessive, X-linked recessive, and *de novo*.

This workflow employed stringent criteria to prioritize the most promising candidate genes for MIS-C, focusing on ultra-rare variants (MAF ≤0.01%) to capture potentially novel and pathogenic mutations. This rigorous filtering minimized false positives and ensured that only significant variants segregating according to expected inheritance patterns were considered.

#### Approach 2: MIS-C monogenic candidate variants prioritization workflow

We adopted a second prioritization workflow focused on a panel of 84 genes associated with MIS-C (**Supplementary Table 2**). This panel was compiled from published studies and consortium data, with gene selections based on genomic variants and differential gene expression studies linked to MIS-C (van der Made et al., 2020; Zhang et al., 2020; Lee et al., 2020; Chou et al., 2021; Solanich et al., 2021; Asano et al., 2021; Abolhassani et al., 2022; Abuhammour et al., 2022; Vagrecha et al., 2022; Reis et al., 2023; Lee et al., 2023).

This workflow prioritized rare exonic variants (MAF ≤1%) and potentially damaging mutations, adapting filtering criteria from Reis et al., 2023. Variants were further evaluated for pathogenicity using the American College of Medical Genetics and Genomics (ACMG) classification (Richards et al., 2015) via Varsome (http://varsome.com) and inheritance patterns obtained from Online Mendelian Inheritance in Man (OMIM: http://www.omim.org).

By adopting a curated gene panel and a more relaxed MAF threshold (≤1%), this approach ensured consistency with similar studies and focused on identifying rare, clinically relevant variants for MIS-C.

### Examining the prioritized candidate genes and variants against the publicly available database on differentially expressed genes in MIS-C versus control individuals

Publicly-available data sets of 1,077 differentially expressed genes between individuals with SARS-CoV-2 associated MIS-C and controls (de Cevins et al., 2021), performed on all peripheral blood mononuclear cells (PBMCs) and on major cell types (monocytes/conventional dendritic cells (cDCs)/plasmacytoid dendritic cells (pDCs), T cells, and B cells), were used to further prioritise our candidate MIS-C genes and variants.

### Integrated analysis of pathways, gene ontology, and protein-protein interactions for MIS-C candidate genes and variants

To elucidate the biological processes and pathways impacted by the prioritised candidate genes and candidate variants linked to MIS-C, we conducted several analyses. These included functional enrichment analysis, protein functional interaction network analysis covering protein-protein, protein-DNA-genetic interactions, and biological pathways, and tissue enrichment analysis. For the analysis of protein functional interaction networks associated with variant genes, we utilized GeneMania (Franz et al., 2018), an online platform. Gene ontology enrichment analysis was performed on genes showing a statistically significant increase in genetic load using WebGestalt (Zhang et al., 2005; Wang et al., 2013). Additionally, we carried out biological process enrichment analysis for variant-associated genes using tools such as DAVID (Shannon et al., 2003) and Enrichr (Kuleshov et al., 2016). Pathway enrichment analysis was conducted using KEGG, Reactome, and WikiPathways databases. The most statistically significant GO terms were visualized using the ggplot2 package in R. Cytoscape software was employed to build the co-expression network.

## Results

### Demographic and clinical characteristics of the MIS-C patients

A total of 24 patients, predominantly of Middle Eastern origin, from Kuwait, with MIS-C were prospectively recruited into the study cohort. The cohort comprised 18 males (75%) and 6 females (25%), with the majority being Arab, including 10 Kuwaiti, 2 Syrian, and 6 Egyptian patients. The remaining patients were of Asian descent, consisting of 4 Indian, 1 Sri Lankan, and 1 Afghan individuals. The mean age ± standard deviation of the participants was 6.35 ± 3.96 years. Most patients, 83.3% (20/24), had evidence of direct SARS-CoV-2 infection, as confirmed by serology or PCR testing.

Clinical manifestations across the cohort included gastrointestinal symptoms in 54.2% (13/24) of patients, skin rashes in 75% (18/24), and neurological complications in 12.5% (3/24) of the cases. Cardiac complications were noted, with 9.5% (2 out of 21 probands with available data) diagnosed with myocarditis, and 25% (6 out of 24) with coronary aneurysm/dilatation. Furthermore, 8.3% (2/24) of the cohort were diagnosed with classic Kawasaki disease, while a significant majority, 83.3% (20/24), were diagnosed with MIS-C or fulfilled the criteria of MIS-C diagnosis. Critical conditions necessitating intensive care were observed in 29.2% (7/24) of the patients. Parental consanguinity was documented in 16.7% (4/24) of cases. The comprehensive clinical characteristics and outcomes for these probands are detailed in **Supplementary Table 1**.

### Results of variant calling using MIS-C monogenic candidate genes approach

In our exome sequencing study of 24 trio families, we identified a total of 115,182 exonic variants. Among these, 53,960 were non-synonymous, 49,804 were synonymous, 821 were stop-gain mutations, and 124 were stop-loss mutations. Additionally, 2,989 frameshift and non-frameshift mutations were identified, along with 497 splice-site variants. The remaining variants were either unknown or labelled as “.”.

In applying candidate genes prioritization workflow to the exonic variants, we successfully identified 55 variants from 53 genes from 20 of the 24 probands (**Table 1**). This set of prioritized variants encompassed 7 *de novo*, 27 X-linked recessive hemizygous, and 21 autosomal recessive homozygous mutations. 51 of these 55 variations were non-synonymous, two were stop-gain and two were splice-altering mutations. Interestingly, 15 probands were found to have multiple prioritized variants. However, a direct comparison of their phenotypic profiles revealed no distinct shared characteristics when compared to the nine probands that did not have multiple prioritized variants.

**Table 1:** Prioritised candidate genes for MIS-C by way of using exome data of the 24 families and using the candidate gene approach.

Proband, Sex	Genes/Variants	Effect	AF (gnomAD)	CADD phred	ClinVar	ACMG	Inheritance
P1, Male	*MTMR8*:NM_017677:c.T709C:p.Y237H	non-synonymous	–	23.7	–	VUS (PP3,PM2,BP1)	X-linked recessive
P2, Male	*KIF18A*:NM_031217:c.C1014G:p.F338L	non-synonymous	–	24	–	Likely Benign (BP1,BP4,PM2)	*De novo*
P3, Male	*DDX60*:NM_017631:c.C2269G:p.P757A	non-synonymous	–	22.8	–	Likely Benign (BP1,BP4,PM2)	Autosomal recessive
P3, Male	*RASSF3*:NM_178169:c.C82A:p.P28T	non-synonymous	–	22.9	–	VUS (PM2,BP4)	*De novo*
P3, Male	*ARHGAP4*:NM_001164741:c.G771A:p.W257X	stop-gain	–	23.5	–	Likely Pathogenic (PVS1,PM2)	X-linked recessive
P3, Male	*EGFL6*:NM_001167890:c.G1429T:p.G477W	non-synonymous	–	33	–	Likely Benign (BP1,BP4,PM2)	X-linked recessive
P3, Male	*RHBDD2*:NM_001346187:c.C323T:p.P108L	non-synonymous	0.00003	33	–	VUS (PM2,BP4)	Autosomal recessive
P3, Male	*TMEM154*:NM_152680:c.G544T:p.E182X	stop-gain	–	37	–	VUS (PM2)	*De novo*
P4, Male	*GAB3*:NM_001282283:c.C1249T:p.P417S	non-synonymous	0.00001	24	–	Likely Benign (BP4,PM2)	X-linked recessive
P4, Male	*ADGRG4*:NM_153834:c.G8248A:p.G2750R	non-synonymous	0.00005	34	–	Likely Benign (BP1,BP4,PM2)	X-linked recessive
P5, Male	*DMD:* NM_004009:c.C2815T:p.R939C	non-synonymous	0.0001	29.2	Conflict reports	Benign (BS2,BP6,BP1)	X-linked recessive
P6, Male	*DNAH3*:NM_001347886:c.A11180G:p.Y3727C	non-synonymous	–	22.2	–	Benign (BS1,BS2,BP4,BP1)	Autosomal recessive
P6, Male	*ADGRG4*:NM_153834:c.A4477G:p.N1493D	non-synonymous	0.0001	22.3	VUS	Benign (BS1,BS2,BP4,BP1,BP3)	X-linked recessive
P6, Male	*PCDH19*:NM_020766:c.C762A:p.N254K	non-synonymous	–	22.7	VUS	VUS (PM1,PM2,BP4)	X-linked recessive
P6, Male	*ABAT:* NM_000663:c.C947T:p.A316V	non-synonymous	0.00001	23.7	–	VUS (PM2,BP4)	Autosomal recessive
P6, Male	*MAP3K15:*NM_001001671:c.G1591A:p.V531I	non-synonymous	–	24.5	–	Likely Benign (BP1,BP4,PM2)	X-linked recessive
P6, Male	*ARMCX2*:NM_177949:c.C7T:p.R3C	non-synonymous	–	32	–	Likely Benign (BP1,BP4,PM2)	X-linked recessive
P6, Male	*NID1*:NM_002508:c.C1166T:p.T389M	non-synonymous	0.0007	32	–	Likely Benign (BP4,BP1,PM2)	Autosomal recessive
P7, Female	*USF2*:NM_003367:c.C403T:p.P135S	non-synonymous	0.0003	22.1	VUS	Benign (BS1,BS2,BP4,BP1)	*De novo*
P7, Female	*P2RY2*:NM_002564:c.C364A:p.L122I	non-synonymous	–	22.2	–	VUS (PM2,BP4)	Autosomal recessive
P7, Female	*POGLUT3*:NM_001363502:c.C1042T:p.P348S	non-synonymous	0.0002	22.3	VUS	Likely Benign (BP4,PM2)	Autosomal recessive
P8, Male	*TBX22*:NM_001303475:c.G782T:p.W261L	non-synonymous	0.000005	22.6	–	Likely Benign (BP4,PM2)	X-linked recessive
P9, Male	*EGFL6*:NM_001167890:c.G1429T:p.G477W	non-synonymous	–	33	–	Likely Benign (BP1,BP4,PM2)	X-linked recessive
P10, Female	*SFRP5*:NM_003015:c.C938T:p.A313V	non-synonymous	0.0002	23.2	–	Likely Benign (BP1,BP4,PM2)	Autosomal recessive
P10, Female	*RADIL:* NM_018059:c.G574A:p.A192T	non-synonymous	0.0002	23.4	–	Likely Benign (BP1,BP4,PM2)	Autosomal recessive
P10, Female	*GBF1*:NM_004193:c.C3026T:p.A1009V	non-synonymous	0.0003	25.1	–	Benign (BS1,BS2,BP4,BP1)	Autosomal recessive
P10, Female	*MIS18BP1*:NM_018353:c.G1033A:p.A345T	non-synonymous	–	25.5	–	Likely Benign (BP4,PM2)	*De novo*
P11, Male	*ARR3*:NM_004312:c.C577T:p.P193S	non-synonymous	0.0002	22.1	Likely Benign	Benign (BS1,BS2,BP1,BP4,BP6)	X-linked recessive
P11, Male	*MCF2*:NM_001171878:c.G2216A:p.R739H	non-synonymous	0.00001	22.1	–	VUS (PM2,PP3,BP1)	X-linked recessive
P11, Male	*MAGEB2*:NM_002364:c.A647G:p.N216S	non-synonymous	0.000005	22.4	VUS	Likely Benign (BP1,PB4,PM2)	X-linked recessive
P11, Male	*ARAF:* NM_001256196:c.G633C:p.Q211H	non-synonymous	0.00001	23.8	–	VUS (PM1,PM2,BP4)	X-linked recessive
P12, Male	*ARR3*:NM_004312:c.C577T:p.P193S	non-synonymous	0.0002	22.1	Likely Benign	Benign (BS1,BS2,BP1,BP4,BP6)	X-linked recessive
P12, Male	*MCF2*:NM_001171878:c.G2216A:p.R739H	non-synonymous	0.00001	22.1	–	VUS (PM2,PP3,BP1)	X-linked recessive
P12, Male	*MAGEB2*:NM_002364:c.A647G:p.N216S	non-synonymous	0.000005	22.4	VUS	Likely Benign (BP1,PB4,PM2)	X-linked recessive
P12, Male	*ARAF:* NM_001256196:c.G633C:p.Q211H	non-synonymous	0.00001	23.8	–	VUS (PM1,PM2,BP4)	X-linked recessive
P13, Male	*ZNF41*:NM_001324147:c.G1082A:p.G361E	non-synonymous	–	24.8	–	Likely Benign (BP1,PB4,PM2)	X-linked recessive
P13, Male	*AHDC1*:NM_001029882:c.C104A:p.P35H	non-synonymous	–	25.2	–	Likely Benign (BP1,PB4,PM2)	*De novo*
P14, Male	*SRPX:* NM_001170752:c.G256A:p.D86N	non-synonymous	0.0001	21.7	–	Benign (BS1,BS2,BP1,BP4)	X-linked recessive
P14, Male	*OBSL1*:NM_015311:c.C2770T:p.R924W	non-synonymous	0.000008	22.1	–	Likely Benign (BP1,PB4,PM2)	*De novo*
P15, Male	*MECP2*:NM_001110792:c.G898A:p.V300M	non-synonymous	0.00009	23.9	Benign	Benign (BS2,BP4,BP6,PM1)	X-linked recessive
P15, Male	*HS6ST2*:NM_001077188:c.948-2A>G	splicing	0.00007	25.6	–	Likely Benign (BS1,BS2,PVS1)	X-linked recessive
P16, Male	*LRRFIP1*:NM_001137550:c.A575G:p.H192R	non-synonymous	0.00004	22	–	Likely Benign (BP1,BP4,PM2)	Autosomal recessive
P16, Male	*DLX3*:NM_005220:c.C376T:p.P126S	non-synonymous	0.00002	22.9	VUS	Likely Benign (BS2)	Autosomal recessive
P16, Male	*ZMYM3*:NM_201599:c.G2219A:p.R740H	non-synonymous	0.000005	34	VUS	VUS (PP2,PM2,BP4)	X-linked recessive
P17, Male	*SMARCA1*:NM_001282874:c.C215T:p.A72V	non-synonymous	0.00002	20.1	–	Benign (BS2,BP4,BP1)	X-linked recessive
P17, Male	*SYTL4*:NM_001370164:c.G962A:p.R321H	non-synonymous	0.00003	34	–	Benign (BS1,BS2,BP4)	X-linked recessive
P18, Female	*LSG1*:NM_018385:c.G665A:p.R222Q	non-synonymous	0.0003	20.9	–	Likely Benign (BP4,PM2)	Autosomal recessive
P18, Female	*NDC1*:NM_001168551:c.T1504C:p.F502L	non-synonymous	0.0006	21.8	–	Benign (BS1,BS2,BP4)	Autosomal recessive
P18, Female	*ABCC10*:NM_001350518:c.A1957G:p.S653G	non-synonymous	0.00007	22.3	–	Likely Benign (BP1,BP4,PM2)	Autosomal recessive
P18, Female	*TRERF1*:NM_033502:c.G3415T:p.V1139L	non-synonymous	0.000003	22.4	–	Likely Benign (BP1,BP4,PM2)	Autosomal recessive
P18, Female	*DCTD:* NM_001351743:c.G528C:p.K176N	non-synonymous	0.000008	22.9	VUS	Likely Benign (BP4,PM2)	Autosomal recessive
P18, Female	*SLC4A5*:NM_133478:c.1268 + 2T>C	splicing	–	25.8	–	Likely Pathogenic (PVS1,PM2)	Autosomal recessive
P19, Male	*APOOL:* NM_198450:c.G190A:p.G64S	non-synonymous	0.0003	23.4	–	Benign (BS1,BS2,BP3)	X-linked recessive
P19, Male	*NAF1*:NM_138386:c.G373A:p.D125N	non-synonymous	0.0007	23.4	Benign	Benign (BS1,BS2,BP1,BP4)	Autosomal recessive
P19, Male	*RHO:* NM_000539:c.T182C:p.V61A	non-synonymous	0.000003	23.6	–	VUS (PM1,PP3,PM2)	Autosomal recessive
P19, Male	*USH2A*:NM_206933:c.C1898T:p.S633L	non-synonymous	0.0002	24.2	Conflict reports	Likely Benign (BP4,PM1,PM2)	Autosomal recessive
P19, Male	*SHROOM2*:NM_001649:c.C623T:p.S208L	non-synonymous	0.00001	25	–	VUS (PM1,PM2)	X-linked recessive
P20, Male	*USP9X*:NM_001039591:c.A5567G:p.E1856G	non-synonymous	–	23.1	–	Likely Benign (BP1,BP4,PM1,PM2)	X-linked recessive
P20, Male	*PRRG1*:NM_001173486:c.T36A:p.N12K	non-synonymous	–	23.9	–	VUS (PM2,BP4)	X-linked recessive
P20, Male	*DMD:* NM_004013:c.G1565A:p.R522Q	non-synonymous	0.0003	24	Benign	Benign (BS2,BP1,BP4,BP6)	X-linked recessive

AF, Allele Frequency, gnomAD; CADD phred, Combined Annotation Dependent Depletion Phred Score; ClinVar, Clinical Variant Database; ACMG, American College of Medical Genetics and Genomics.

We annotated the above-mentioned 55 variants using the VEP tool against ClinVar entries (https://www.ncbi.nlm.nih.gov/clinvar/). This revealed that 13 of the 42 variants were reported in ClinVar database. Of those 13 variants, seven (namely *ADGRG4*:p.N1493D, *DLX3*:p.P126S, *PCDH19*:p.N254K, *MAGEB2*:p.N216S, *USF2*:p.P135S, *RHO*:p.V61A, and *DCTD*:p.K176N) were reported as ‘uncertain significance’, four (namely *NAF1*:p.D125N, *MECP2*:p.V300M, *ARR3*:p.P193S and *DMD*:p.R522Q) were reported as ‘benign or likely benign’, and the remaining two (namely *USH2A*:p.S633L and *DMD*:p.R939C) had conflicting pathogenicity reports. More than one variant from same gene were seen in two instances: *ADGRG4*:p.G2750R and *ADGRG4*:p.N1493D in P4 and P6, respectively, and *DMD*:p.R939C and *DMD*:p.R522Q in P5 and P20, respectively (see **Table 1**). Further, we noted a recurrence of specific X-linked recessive variants (namely *EGFL6*:p.G477W, *ARAF*:p.Q211H, *ARR3*:p.P193S, *MAGEB2*:p.N216S, and *MCF2*:p.R739H) in male probands.

### Results of variant calling using MIS-C candidate variants approach

In a complementary approach focused specifically on MIS-C monogenic candidate genes, our targeted analysis of exome sequence data identified a total of 537 variants located within the 84-gene panel for MIS-C. Among these, 534 were exonic variants and 3 were splicing variants. The exonic variants included 204 nonsynonymous, 3 frameshift deletions, 2 nonframeshift deletions, 2 stopgain mutations, 3 stoploss mutations, and 235 synonymous. The remaining 85 variants were classified as unknown. Of these, 121 variants were deemed to have potential functional consequences based on their nonsynonymous, frameshift, splicing, or stop-loss/gain effects.

By way of applying the MIS-C candidate variants prioritization workflow to the identified variants, we prioritised 33 rare predicted deleterious heterozygous variants, spread across 19 unique genes. These 33 prioritised variants were seen in 19 of the 24 probands (**Table 2**).

**Table 2:** Prioritised candidate variants for MIS-C using 24 probands exome data by way of using gene panel associated with MIS-C and applying the candidate variant approach.

Gene	Variant	Effect	AF (gnomAD)	ClinVar	CADD phred	Patients	ACMG	Inheritance pattern (OMIM)
*AP3B1*	NM_003664:c.3023_3025del:p.1008_1009del	non-frameshift	0.009	Benign	–	P4, P18	Benign (PM4,BA1,BP6)	AR
*ATM*	NM_001351834:c.C1810T:p.P604S	non-synonymous	0.003	Conflict reports	24.5	P5, P13	Benign (BS1,BS2,BP4,BP6,BP1)	AR/AD
*C6*	NM_001115131:c.C62T:p.A21V	non-synonymous	0	VUS	21.3	P3, P5	Likely Benign (PM2,BP1,BP4)	AR
*LY9*	NM_002348:c.510_512del:p.170_171del	non-frameshift	–	–	–	P23	Benign (PM4,BS1,BS2,BP4)	AR
*LY9*	NM_002348:c.1052_1053del:p.H351fs	frameshift	0	Risk factor	–	P13	VUS (PM2)	AR
*IFIH1*	NM_022168:c.2016delA:p.T672fs	frameshift	0	Conflict reports	–	P11	VUS (PVS1,PM2,BP6)	AR/AD
*DOCK8*	NM_203447:c.C3022T:p.R1008W	non-synonymous	0.001	Benign	35	P23	Benign (PM2,BP6,BP4,BP1)	AR
*PSTPIP1*	NM_001321137:c.C877T p.R293C	non-synonymous	0	VUS	34	P4	Likely Benign (PP3,PS3,BS2,BP1)	AD
*ATM*	NM_001351834:c.G1516T:p.G506C	non-synonymous	0	Conflict reports	33	P24	Likely Benign (PM2,BP6,BP1,BP4)	AR/AD
*ATM*	NM_001351834:c.G3257A:p.R1086H	non-synonymous	0	VUS	31	P16	VUS (PM2,BP1)	AR/AD
*NOD2*	NM_001293557:c.G2641C:p.G881R	non-synonymous	0.01	Conflict reports	29.9	P9	Benign (PP5,BS1,BS2,BP4,BS3,BP6)	AD
*NLRP12*	NM_001277129:c.C850T:p.R284X	stop-gain	0	Pathogenic	29.6	P8	Likely Benign (PVS1,PS3,BS1, BS2)	AD
*IFIH1*	NM_022168:c.2807 + 1G>A	splicing	0.007	Conflict reports	28.5	P20	Likely Benign (PVS1,PS3,BS2,BS3)	AR/AD
*DOCK8*	NM_203447:c.G2554A:p.V852M	non-synonymous	0	VUS	28.2	P16	Likely Benign (PM2,BP1,BP4)	AR
*ATM*	NM_001351834:c.G3449C:p.R1150T	non-synonymous	0	Conflict reports	27.6	P19	Likely Benign (PM2,BP4,BP6,BP1)	AR/AD
*RNASEL*	NM_021133:c.G523T:p.A175S	non-synonymous	0	–	27	P14	VUS (PP3,PM2,BP1)	AR/AD
*DCLRE1C*	NM_001033855:c.C251G:p.S84C	non-synonymous	0	–	26.4	P9	Likely Benign (PM2,BP1,BP4)	AR
*C6*	NM_000065:c.2381 + 2T>C	splicing	0.002	Conflict reports	26.4	P21	Pathogenic (PVS1,PP5,PM2)	AR
*LRBA*	NM_001199282:c.T2466G:p.I822M	non-synonymous	0.001	–	25.3	P15	Likely Benign (PM2,BP4,BP6,BP1)	AR
*CR2*	NM_001877:c.G1676A:p.G559E	non-synonymous	0.001	Conflict reports	24.7	P13	Likely Benign (PM2,BP4,BP6,BP1)	AR
*PSTPIP1*	NM_001321137:c.C398T:p.T133M	non-synonymous	0.002	Conflict reports	24.6	P17	Benign (BP6,BS1,BS2,BP4,BP1)	AD
*LYST*	NM_001301365:c.C5048T:p.A1683V	non-synonymous	–	–	24.6	P13	VUS (PM2,PP3,BP1)	AR
*UNC13D*	NM_199242:c.T1039C:p.S347P	non-synonymous	0	Uncertain significance	24.3	P7	VUS (PM2)	AR
*IRAK3*	NM_001142523:c.A328G:p.I110V	non-synonymous	0.001	.	24.2	P19	Likey Benign (PM2,BP4,BP1,BP6)	AR
*TTC7A*	NM_020458:c.G563T:p.R188L	non-synonymous	0.003	Benign	23.9	P15	Likely Benign (BS1,BS2,BP4,BP6,BP1)	AR
*CR2*	NM_001877:c.G2856C:p.Q952H	non-synonymous	0.001	Conflict reports	23.8	P24	Benign (BS1,BS2,BP4,BP6,BP1)	AR
*STING1*	NM_001367258:c.G218T:p.G73V	non-synonymous	0	Conflict reports	22.5	P18	Likely Benign (PM2,BP4)	AR/AD
*DOCK8*	NM_203447:c.C4346T:p.S1449L	non-synonymous	0	Conflict reports	22.5	P18	Likely Benign (PM2,BP4,BP1,BP6)	AR
*LYST*	NM_001301365:c.A10510G:p.I3504V	non-synonymous	–	–	22.2	P4	Likely Benign (PM2,BP4,BP1)	AR
*LIG4*	NM_002312:c.A303T:p.R101S	non-synonymous	–	–	22	P12	Likely Benign (PM2,BP4)	AR
*IFIH1*	NM_022168:c.A1046G:p.K349R	non-synonymous	0.003	Benign	21.8	P15	Benign (BS1,BS2,BP4,BP6,BP1)	AR/AD
*TTC7A*	NM_020458:c.G2258A:p.R753Q	non-synonymous	0.001	Likely benign	21.5	P3	Likely Benign (PM2,BP4,BP6,BP1)	AR
*IRAK3*	NM_001142523:c.T989C:p.M330T	non-synonymous	0.001	–	20.8	P19	Likely Benign (PM2,BP4,BP1,BP6)	AR

AF, Allele Frequency, gnomAD; ClinVar, Clinical Variant Database; CADD phred, Combined Annotation Dependent Depletion Phred Score; ACMG, American College of Medical Genetics and Genomics; OMIM, Online Mendelian Inheritance in Man.

We annotated the 33 variants prioritized through the gene panel approach using the VEP tool against the ClinVar database. This classified one variant, namely *NLRP12*:p.R284X, as ‘Pathogenic’ and another variant, namely *LY9*:p.H351fs, as ‘Risk factor ‘. We encountered 5 variants (from genes such as *C6* and *DOCK8*) annotated as of ‘Uncertain significance’. Significantly, 26 of the 33 variants were not reported in ClinVar, highlighting the potential for reporting novel genetic associations with MIS-C. Using ACMG guidelines, one variant, namely *C6*:c.2381 + 2T>C, was classified as ‘Pathogenic’, six variants, namely *LY9*:p.H351fs, *IFIH1*:p.T672fs, *ATM*:p.R1086H, *RNASEL*:p.A175S, *LYST*:p.A1683V, and *UNC13D*:p.S347P, were classified as ‘Variants of Unknown Significance’ (VUS), and the remaining variants were classified as either ‘Benign’ or ‘Likely Benign’.

Furthermore, our findings underscore the recurrence of specific variants in multiple probands, potentially pinpointing causative candidate variants or delineating their relevance in the pathogenesis of MIS-C. For example, the *ATM*:p.P604S variant appeared in probands P5 and P13 and the *C6*:p.A21V variant appeared in probands P3 and P5. Additionally, the *AP3B1* gene harbouring the p.1008_1009del variant was identified in probands P4 and P18. It is worth noting that the probands P5 and P13, sharing the *ATM* gene variant, as well as the P4 and P18 probands, sharing the *AP3B1* gene variant, were presented with ‘Critical’ severity of MIS-C symptoms and some overlapping clinical features. This observation may suggest a contributory role for these genetic variants in determining the severity of MIS-C presentations.

### Differential expression of MIS-C candidate genes and variants

The prioritised genes were examined against significant results from the study of de Cevins et al. (2021); this study examined differentially expressed genes in MIS-C (CoV-2+) afflicted children compared to control children in different cell types such as PBMCs (200 genes), Monocytes/cDCs/pDCs (653 genes), T cells (177 genes) and B cells (423 genes). Of the 1,077 unique differentially expressed genes analysed for overlap with prioritized monogenic candidate genes in the gene panel approach, three genes—*DDX60*, *TMEM154*, and *RASSF3*—were found to be common. Notably, all three genes were prioritized from proband P3 (**Table 3**). Additionally, five genes—*LY9*, *IFIH1*, *DOCK8*, *DCLRE1C*, and *LYST*—were identified as common among the prioritized monogenic candidate genes using the family trio approach, observed in nine patients (**Table 3**).

**Table 3:** Prioritised MIS-C genes common with differentially expressed genes in MIS-C (CoV-2+) afflicted children compared to control children in different cell types (de Cevins et al., 2021).

Gene	Avg_logFC	Adjusted p value	Cell type	Proband
Prioritised monogenic candidate genes				
*DDX60*	0.38	0	All PBMC	P3
	0.48	3.88E-209	Monocytes/cDCs/pDCs	
	0.38	1.33E-255	T cells	
	0.33	1.53E-81	B cells	
*TMEM154*	0.32	1.41E-150	All PBMC	
	0.33	7.68E-25	Monocytes/cDCs/pDCs	
	0.40	2.13E-61	B cells	
*RASSF3*	0.33	4.38E-38	Monocytes/cDCs/pDCs	
Prioritised monogenic candidate variants				
*LY9*	-0.33	2.22E-09	B cells	P13,P23
*IFIH1*	0.40	5.20E-83	Monocytes/cDCs/pDCs	P11,P15,P20
*DOCK8*	0.29	2.04E-142	All PBMC	P16,P18,P23
	0.55	3.68E-93	Monocytes/cDCs/pDCs	
*DCLRE1C*	0.28	1.70E-29	B cells	P9
*LYST*	0.55	6.51E-83	Monocytes/cDCs/pDCs	P4,P13

PBMCs, Peripheral Blood Mononuclear Cells; cDCs, Conventional Dendritic Cells; pDCs, Plasmacytoid Dendritic Cells.

### Analysis of functional enrichment, examination of pathways, and investigation of protein-protein interactions

**Figure 1:**
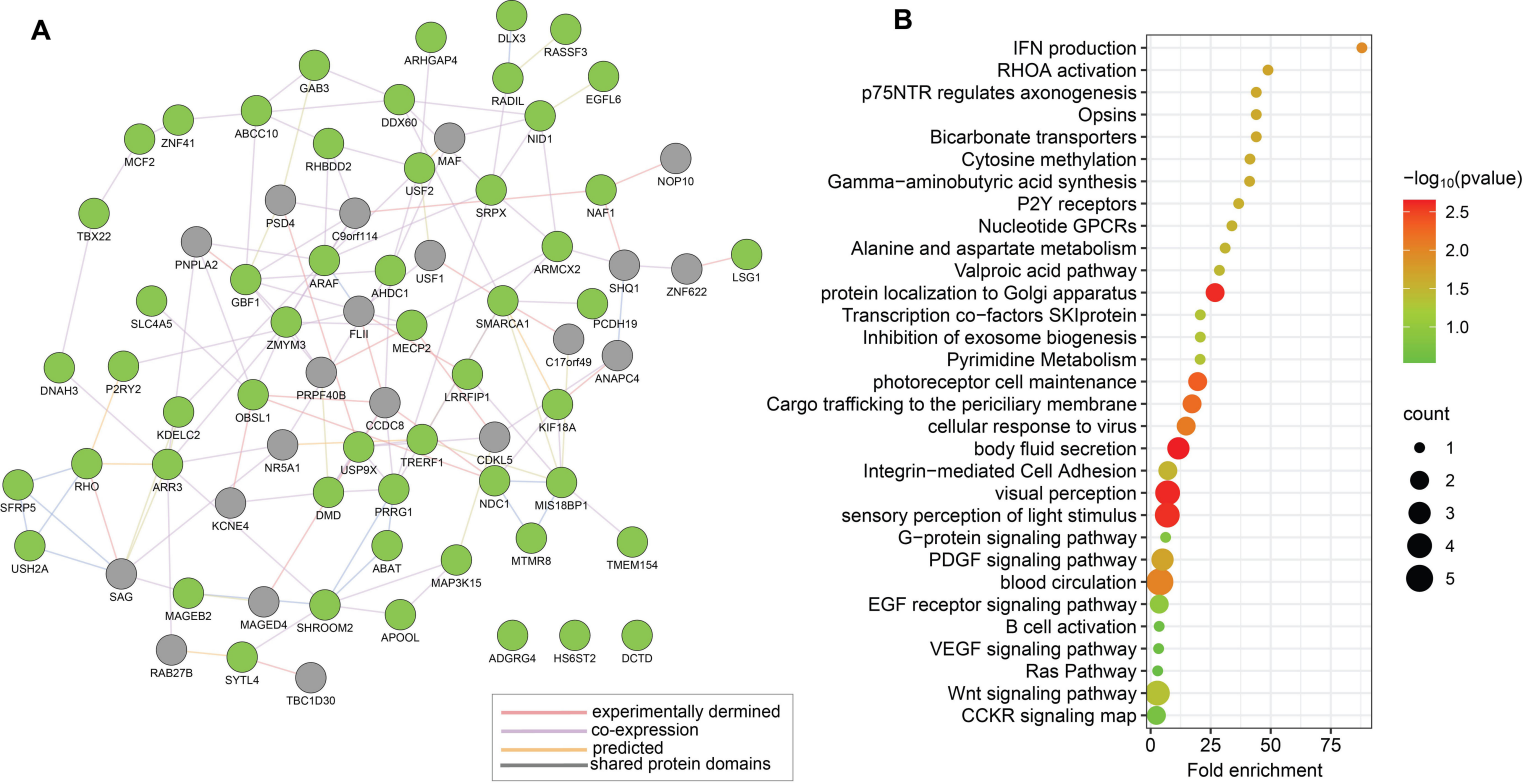
Functional relationships between the 53 genes prioritised by candidate genes approach (Approach 1) for MIS-C. **(A)** Schematic illustration of a PPI network based on experimental evidence and expert-curated databases by using GeneMANIA. The network features a subset of 53 candidate genes for MIS-C, represented as green nodes. Grey nodes represent genes that interact with the candidate genes, as supported by experimental evidence and database curation. **(B)** Representative results from enrichment analyses of the prioritized 53 candidate genes for MIS-C using gene ontology terms for biological process and KEGG pathways. Each identified category shows enrichment, including the -log_10_ of the statistical enrichment P-value from the DAVID database where maximum enrichment was observed, alongside the count of involved genes. The dot size reflects the number of genes enriched in each category.

Upon investigating the regulatory networks of the identified 53 unique genes from the candidate gene approach for MIS-C (as presented in **Table 1**) using Protein-protein Interaction (PPI) networks and protein-DNA-genetic interactions networks of genes constructed by GeneMANIA, we identified relationships between genes that could indicate possible disruptions in signalling pathways in MIS-C (**Figure 1A**). Functional enrichment analysis of these genes indicated that the candidate genes mainly enriched biological pathways, such as IFN production, Rho activation, Wnt, VEGF, EGFR signalling pathways, and visual perception, relevant to MIS-C symptoms (**Figure 1B**).

**Figure 2:**
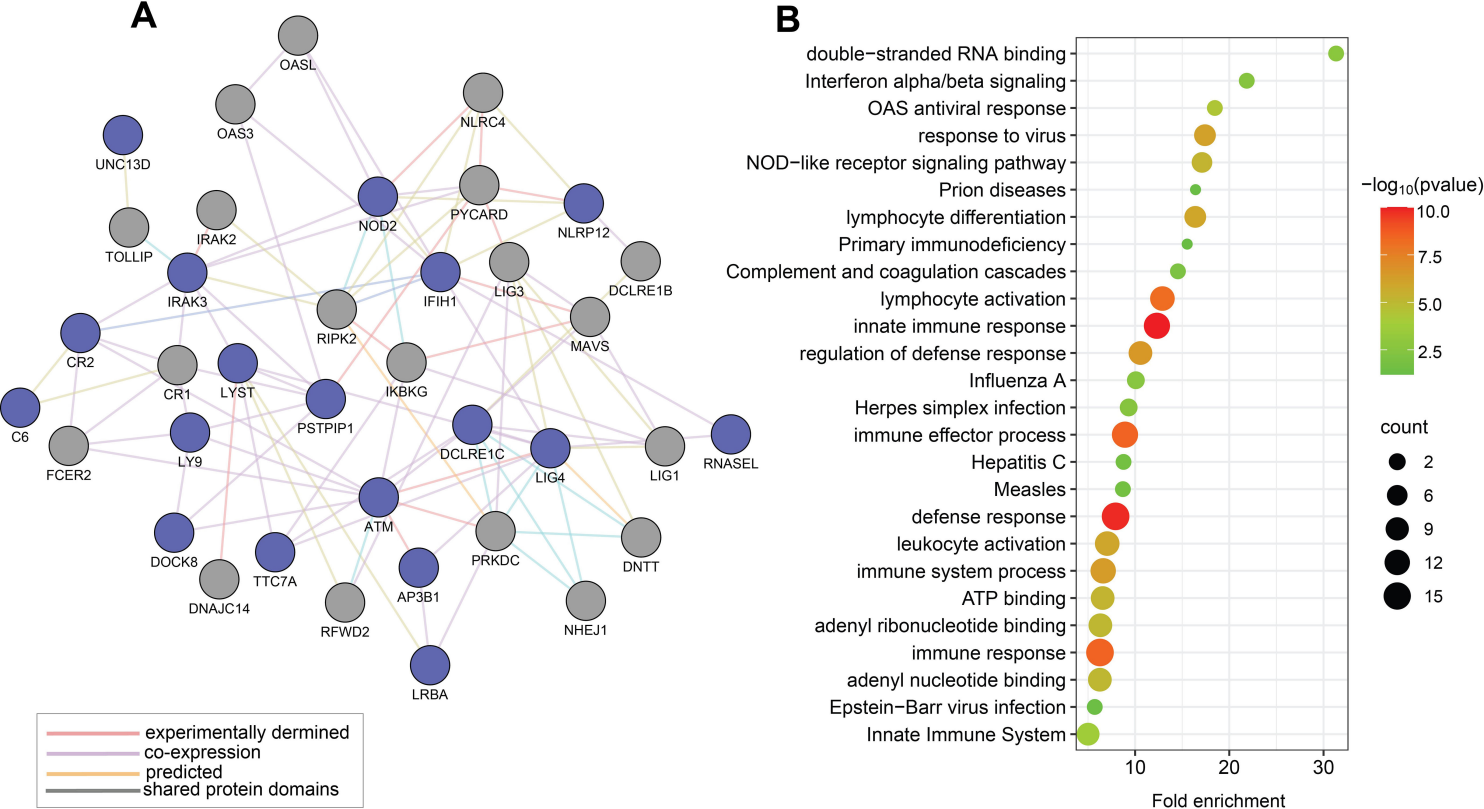
Functional relationships of the 19 genes prioritised by the candidate variants approach for MIS-C (Approach 2). **(A)** This map displays a PPI network of MIS-C-associated genes with prioritized rare candidate variants, represented by blue nodes, and their interacting genes derived from experimental data and expert-curated databases, depicted in grey nodes. **(B)** Representative outcomes from enrichment analyses on selected rare candidate variants in genes linked to MIS-C using gene ontology terms for biological processes and KEGG pathways. Each identified category was enriched, including the -log_10_ of the statistical enrichment P-value from the DAVID database where the enrichment and the respective gene counts were most notable. The dot size reflects the count of genes enriched in each category.

Results of similar analyses on the 19 unique genes identified through the candidate variants approach (depicted in **Table 2**) are presented in **Figure 2**. PPI network of these 19 genes is presented in **Figure 2A** with the 19 genes shown as blue nodes, and their interacting genes (supported by experimental evidence and database curation) shown as grey nodes. **Figure 2B** illustrates the functional enrichment analysis that identifies relevant pathways, including viral response, immune response, inflammation, lymphocyte activation, interferon signalling, and the innate immune system.

**Figure 3:**
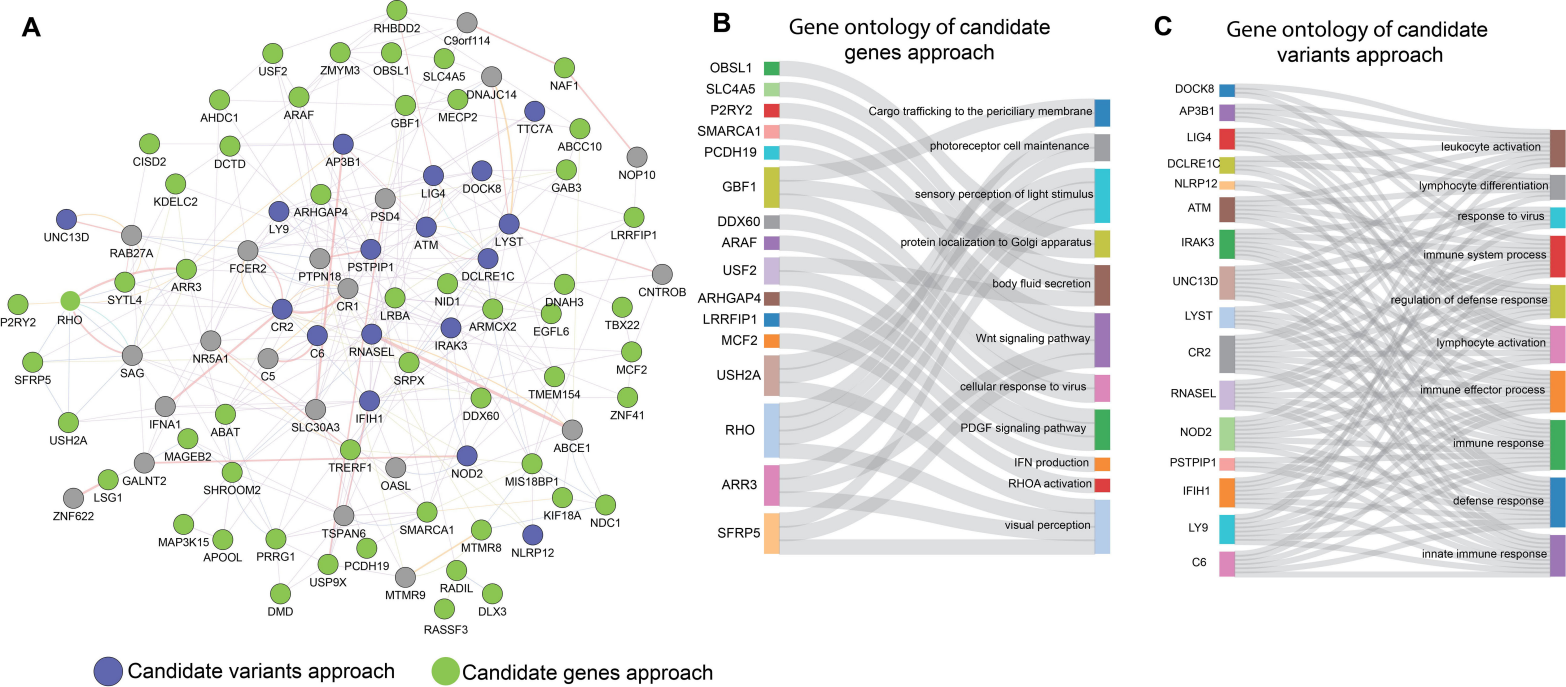
Functional relationships between the two prioritising approaches of candidate genes and candidate variants for MIS-C. **(A)** PPI Networks based on experimental evidence and expert-curated databases. Green node represents the genes with candidate variants identified in our study. Blue colour nodes represent the genes previously reported for MIS-C. **(B, C)** depict Sankey diagrams illustrating the functional relationships between the candidates for MIS-C; **(B)** the prioritised candidate genes approach and **(C)** the prioritised candidate variants approach via biological processes that enrich them according to GO functional enrichment analysis.

To better understand the potential interactions and collective impact of genes identified through the two prioritization approaches for MIS-C, we merged the genes from both analyses into a set of 72 unique genes. Using the GeneMANIA database, we constructed a PPI network consisting of 93 nodes interconnected by 333 edges (**Figure 3A**). This network highlighted significant connections between the genes identified by both the approaches, suggesting they share regulatory pathways relevant to the pathogenesis of MIS-C.

Further analysis was conducted individually on each set of prioritized genes, as depicted in the Sankey diagrams (**Figures 3B, C**). These diagrams demonstrate the functional and biological processes enriched in each approach, including immune response, inflammation, and response to viral pathogens. The overlap of these biological processes in both data sets confirms that both prioritization methods effectively capture the key aspects of the phenotype associated with MIS-C. This phenotype is typically triggered by a viral infection like COVID-19 and manifests as MIS-C. The consistency across both methods underscores their effectiveness in identifying genes that are crucial to the development of MIS-C.

**Figure 4:**
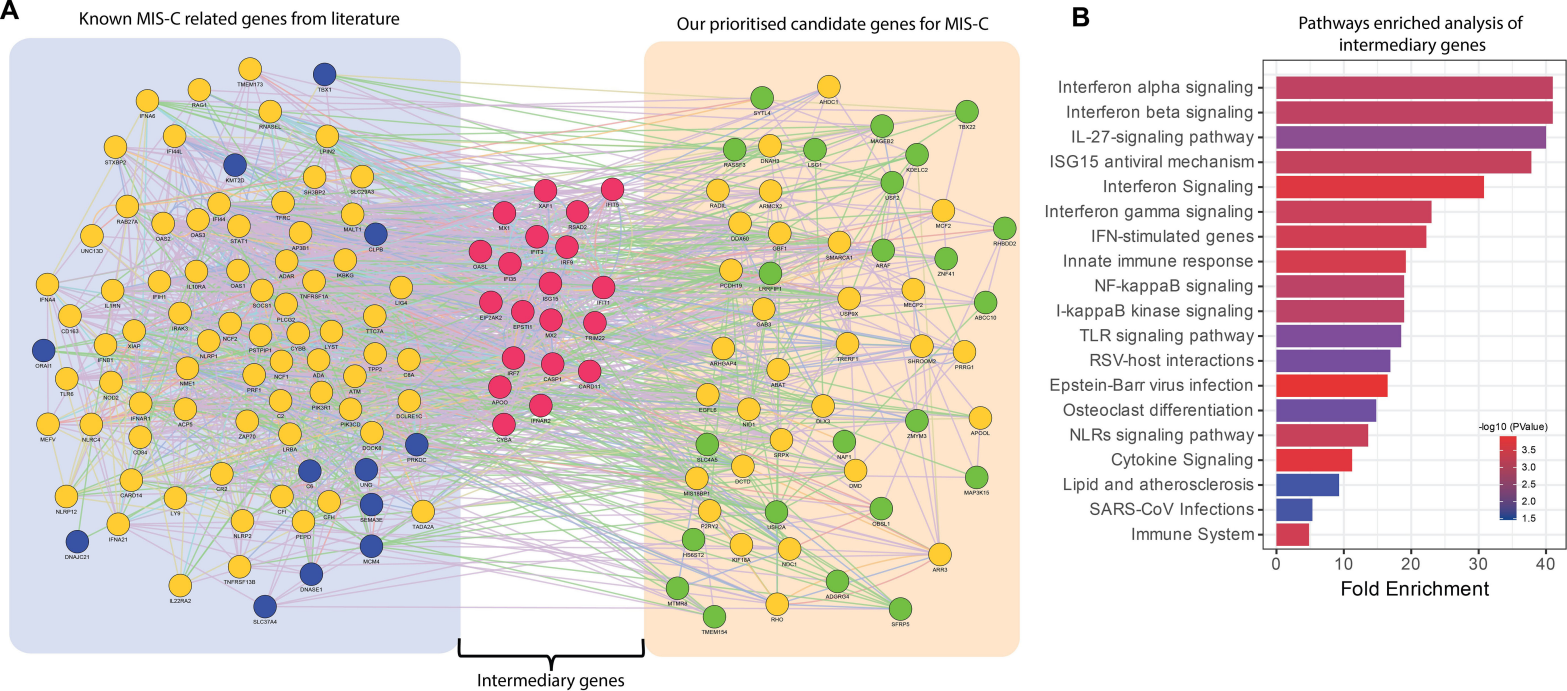
Functional relationships of intermediary genes forming connections between the known genes in MIS-C gene panel and the genes from the prioritized candidate gene approach. **(A)** PPI networks based on experimental evidence and expert-curated databases from GeneMANIA. The left cluster represents genes reported in the literature, known as the MIS-C gene panel (blue nodes). The right cluster shows the prioritized candidate genes identified in our study (green nodes). The middle cluster displays the intermediary genes (red nodes) bridging the two groups of gene sets. Yellow nodes represent genes that interact with both prioritized candidate genes and the MIS-C gene panel. **(B)** Bar plots showing the significant biological processes and pathways of intermediary genes. The x-axis represents fold enrichment, with significance indicated by order and colour trend (P < 0.05).

While the 53 candidate genes prioritized for MIS-C by candidate gene approach (Approach 1) did not overlap with the established 84-gene panel for MIS-C, we attempted to explore the possibility of existence of potential indirect relationships via intermediary genes and to further assesses whether these intermediary genes are functionally relevant to the pathogenesis of MIS-C. This approach would enable us to consider the possibility that, despite the absence of direct gene overlap, interconnected pathways could still play a crucial role in disease mechanisms. To investigate this, we constructed extended PPI networks by way of including both the sets of genes (i.e. the 53 genes derived by the candidate gene approach (Approach 1) and the 84 genes from MIS-C gene panel), aiming to identify intermediary genes mediating secondary connections between the genes. This exercise resulted in 20 intermediary genes (**Figure 4A**). We conducted pathway enrichment analyses on these intermediary genes using the Gene Ontology and KEGG pathway databases (**Figure 4B**). Our findings indicated that several of these intermediary genes are significantly involved in biological pathways critical for immune response, inflammation, and cell signalling processes linked to MIS-C, such as interferon, TLR, NF-kappa beta, cytokine signalling pathway and responses to SARS-CoV infection. This suggests that while there is no direct overlap between the identified candidate genes and the MISC-gene panel, the regulatory networks involving intermediary genes may still significantly influence the pathogenesis of MIS-C.

## Discussion

In our study, we employed two distinct monogenic approaches to elucidate the genetic underpinnings of MIS-C in a predominantly Arab cohort, each proposed different assumptions about inheritance patterns and implications for disease expression.

In our investigation of candidate genes for MIS-C, we adopted a unique methodology by analysing family trios, hypothesizing that heterozygosity in parents could lead to homozygosity or *de novo* mutations in the probands. This approach enabled us to prioritize 53 unique genes, distinguishing our findings from those of broader genomic scans and studies involving control groups, such as those conducted by Chou et al. (2021); Abolhassani et al. (2022); Abuhammour et al. (2022); Gelzo et al., 2022; Santos-Rebouças et al. (2022), and Reis et al. (2023). While these studies provide valuable insights through comparisons across general populations or with COVID-19 positive control children, our targeted exploration within familial settings offers a complementary perspective, enhancing our understanding of the genetic architecture of MIS-C with greater precision.

Following this approach, our functional enrichment analysis and pathway interrogations highlighted key biological processes and pathways associated with these candidate genes (**Figure 1**). Notably, the identified pathways—such as those involved in IFN production, Rho activation, and EGFR signalling—align with a potential state of inflammation that may persist excessively, likely triggered by a COVID-19 infection. This excessive inflammatory response could potentially precipitate the onset of MIS-C, suggesting a critical role for these pathways in the disease’s pathogenesis.

In the candidate genes approach, we observed hemizygous variants in male probands, including recurrent ones such as *EGFL6*:p.G477W, *ARAF*:p.Q211H, *ARR3*:p.P193S, *MAGEB2*:p.N216S, and *MCF2*:p.R739H, alongside different variants in genes like *DMD* and *ADGRG4*, across multiple individuals. These findings suggest a genetic predisposition to MIS-C. Specifically, the *EGFL6* gene is associated with obesity-related inflammation in children (Landgraf et al., 2022), and the *DMD* gene with muscle inflammation (Bez Batti Angulski et al., 2023). Variants in *ARAF*, *ARR3*, *MAGEB2*, and *MCF2* were also found in the same two families presenting identical clinical symptoms. Except for the benign variants *ARR3*:p.P193S and *DMD*:p.R522Q, and the variant *DMD*:p.R939C with conflicting pathogenicity reports, the clinical significance of the other variants remains either uncertain or unreported in ClinVar database. While other prioritized genes from single families show no direct link to MIS-C, this variability could indicate the heterogeneity of this condition. *DDX60* is involved in the antiviral immune response (Miyashita et al., 2011), and the *TMEM154* gene is implicated in viral susceptibility in animals (Heaton et al., 2012). Importantly, both these two genes were observed to be differentially expressed between MIS-C and control children (de Cevins et al., 2021), suggesting their roles in immune regulation and inflammatory response to viral infections.

Our candidate variants approach for MIS-C aligns with pioneering efforts by other groups (Chou et al., 2021; Abolhassani et al., 2022; Abuhammour et al., 2022; Santos-Rebouças et al., 2022; Reis et al., 2023) by focusing on exome data from probands to delineate rare deleterious variants in a population which accommodate incomplete penetrance inheritance in immune and inflammation-related genes that is crucial for MIS-C development. This prioritised approach led to the identification of 33 rare exonic variants with predicted deleterious variants across 19 unique genes known to be associated with MIS-C.

The pathway enrichment analysis performed on the prioritized variants within the known MIS-C gene panel revealed significant involvement in pathways crucial for immune response, viral defence, and inflammation—all key components of MIS-C symptomatology (**Figure 2**). This analysis highlights the relevance of these genes and their pathways in the pathogenesis of MIS-C, thereby reinforcing the validity of the gene panel in identifying causative variants essential for accurate diagnostics.

Following the ACMG guidelines, we identified the splice site variant *C6*:c.2381 + 2T>C in the complement component 6 gene, classified as pathogenic, in one proband from our study cohort. The gene has been implicated in MIS-C development through its role in immune system regulation (Santos-Rebouças et al., 2022; Reis et al., 2023). Gelzo et al. (2022) described this variant in a male MIS-C patient, associating it with severe immune dysregulation, which highlights its contribution to the hyperinflammatory response observed in the syndrome. Another non-synonymous variant (p.A21V) in *C6* gene was prioritised in two probands. However, it is annotated as ‘likely benign’ in ACMG classifications and ‘variant of unknown significance’ in ClinVar.

Moreover, we identified six variants with the annotation of ‘variant of unknown significance’ by ACMG guidelines in 6 genes: *IFIH1*, *LYST*, *UNC13D*, *RNASEL*, *ATM* and *LY9*. One of these, a frameshift variant *IFIH1*:p.T672fs, is involved in the induction of type 1 interferons and proinflammatory cytokines and a candidate gene for MIS-C (Abuhammour et al., 2022; Santos-Rebouças et al., 2022; Vagrecha et al., 2022; Reis et al., 2023). This variant was also described for MIS-C proband by another study (Abuhammour et al., 2022). Additionally, we identified two unreported variants in *IFIH1* for MIS-C; a splice variant c.2807 + 1G>A that is classified as ‘likely benign’ in ACMG classifications and had conflicting reports in ClinVar, and a non-synonymous variant p.K349R that is classified as ‘benign’ in ACMG classifications and ClinVar annotations. Another non-synonymous variant of unknown significance in the *LYST* gene (p.A1683V), which is autosomal recessive monogenic gene for systemic inflammatory syndrome (Schulert et al., 2016), and a potential combination of heterozygous mutations in *LYST* in children with viral infection can lead to MIS-C (Vagrecha et al., 2022). We identified another non-synonymous variant in *LYST*:p.I3504V annotated as ‘likely benign’ in ACMG classifications. Another systemic inflammatory response syndrome gene (Song et al., 2022) and candidate gene for MIS-C (Vagrecha et al., 2022) is *UNC13D*, from which we prioritised a non-synonymous variant, namely p.S347P, annotated as ‘variant of unknown significance’ in both the ACMG classifications and ClinVar annotations. *ATM* is a gene involved in autoimmunity (Gelzo et al., 2022) from which we prioritised a ‘variant of unknown significance’ variant, namely *ATM*:p.R1086H, annotated as ‘variant of unknown significance’ in ACMG classifications. We also identified another non-synonymous variant (benign as per ACMG classification) in *ATM*, namely *ATM*:p.P604S, in multiple probands. We also prioritized the *RNASEL* variant p.A175S, a heterozygous non-synonymous variant annotated as ‘variant of unknown significance’ in ACMG classifications. *RNASEL* plays a key role in antiviral defence, by being involved in IFN-stimulated pathways and cytokine signalling. Lee et al. (2023) linked its homozygous variants to MIS-C, suggesting that our heterozygous variant finding could influence susceptibility to this condition.

The last variant, annotated as ‘variant of unknown significance’ in ACMG classifications but as ‘risk factor’ for MIS-C in ClinVar is *LY9*:p.H351fs. Abuhammour et al. (2022) have previously reported this variant in individuals with MIS-C. *LY9* is involved in the IL-2 signalling pathway and is essential for modulating immune responses and activating lymphocytes. In addition, we have identified a non-frameshift deletion in the *LY9* gene, namely p.170_171del, which has not been reported in ClinVar and is categorized as benign in ACMG classifications.

Although variants classified as benign or likely benign according to ACMG criteria typically do not suggest a disease association, they may still hold potential for novel genetic links with MIS-C. This potential arises from observations by other research groups, who have prioritized variants within the same genes, even when not all variants have been evaluated using ACMG guidelines. Such genes, including *NLRP12*, *DCLRE1C*, *IRAK3*, *PSTPIP1*, *NOD2*, *LRBA*, *CR2*, *AP3B1*, and *DOCK8* (Abolhassani et al., 2022; Abuhammour et al., 2022; Reis et al., 2023; Gelzo et al., 2022; Vagrecha et al., 2022), are also noted for their differential expression in MIS-C versus control groups (**Table 3**). For instance, the *NLRP12*:p.R284X variant, while classified as likely benign by ACMG, is labelled as pathogenic in ClinVar for the rare monogenic autosomal dominant condition of familial cold autoinflammatory syndrome 2 in childhood (Wang, 2022). This discrepancy underscores the complexity of variant classification and the potential for re-evaluation in the context of MIS-C.

To gain a comprehensive understanding of the monogenic determinants of MIS-C, we integrated the findings from our two approaches (namely the candidate gene approach and the candidate variant approach) into a unified analysis (**Figure 3**). This integration revealed significant connections and shared regulatory pathways between the two sets of resultant genes, emphasizing the consistency between the genes identified through each of the two approaches. Such a convergence highlights the crucial roles of immune response, inflammation, and viral pathogenesis in the development of MIS-C, justifying the use of these approaches in parallel to capture the multifaceted nature of the disease.

To further substantiate the relevance of the 53 candidate genes identified for MIS-C by the candidate variant approach, we utilized a protein-protein interaction network analysis to explore their relationships with the established 84-gene MIS-C panel (**Figure 4**). Despite the absence of direct overlap between these two gene sets, our analysis identified intermediary genes that functionally connect them, significantly enriching pathways critical for immune response, inflammation, and cell signalling. This demonstrates that these intermediary genes bridge the candidate and known gene sets, playing pivotal roles in MIS-C pathogenesis through shared biological pathways.

Our dual prioritization approach for identifying candidate genes and variants did not yield conclusive results for some probands, highlighting several important limitations of our study. The diagnostic yield of exome sequencing for monogenic disorders, which stands at approximately 25-58%, suggests that many causative variants may evade detection with this method alone (Dillon et al., 2018). In addition, we did not focus on compound heterozygous variants due to the nascent state of candidate gene identification for MIS-C. Moreover, the absence of prioritized variants in certain probands suggests a need for more comprehensive genetic analysis techniques, such as genome sequencing, that have a higher diagnostic yield and coverage range compared to exome sequencing (Nurchis et al., 2023). Our findings may also be influenced by factors such as variant frequencies exceeding our predefined thresholds, *in silico* predictions of variants being benign or tolerated, absence of COVID-19 positive control group, or the presence of large deletions or insertions, copy number variations, and epigenetic modifications (Davalos et al., 2022). Given the emergent and severe nature of MIS-C, and its overlap with other immunological conditions that can complicate prognosis and diagnosis, as well as the provisional status and incomprehensiveness of the associated gene panel, we proceed with caution. The genes and variants identified in this study are currently proposed to be associated with MIS-C, without a clear mechanistic link to its pathogenesis. This necessitates additional functional genomics studies to validate these associations and to further explore their roles in the disease. Additionally, confirming the findings of our study across cohorts of diverse ethnicities, while accounting for genetic differences among individuals, could enhance the robustness of our results.

In conclusion, our study underscores the significant influence of genetic predispositions, particularly through monogenic mechanisms, on the risk and presentation of MIS-C post-COVID-19. By employing dual approaches to identify candidate genes and variants, we have highlighted potential monogenic origins of MIS-C among children of primarily Arab descent. Future research should focus on confirming these preliminary associations and detailing their mechanistic contributions, thereby aiding in the development of targeted therapeutic strategies.

## Data Availability

The data analysed in this study pertains to minor children and hence the data cannot be made publicly available. However, requests for data may be addressed to HA at hessa.alkandari@dasmaninstitute.org.

## Supplementary material

Supplementary Table 1 Demographic and Clinical Characteristics of the MIS-C Patients.

Supplementary Table 2 MIS-C associated genes panel.
